# Sepsis induces the cardiomyocyte apoptosis and cardiac dysfunction through activation of YAP1/Serpine1/caspase-3 pathway

**DOI:** 10.1515/med-2024-1018

**Published:** 2024-09-17

**Authors:** Xueyuan Long, Yanpeng Yang, Ke Zhou

**Affiliations:** Department of Cardiovascular Medicine, Chongqing University Central Hospital, Chongqing, 400014, China

**Keywords:** sepsis, cardiomyocyte apoptosis, cardiac dysfunction, YAP1, Serpine1, caspase-3

## Abstract

**Background:**

Sepsis triggers myocardial injury and dysfunction, leading to a high mortality rate in patients. Cardiomyocyte apoptosis plays a positive regulatory role in septic myocardial injury and dysfunction. However, the mechanism is unclear.

**Methods:**

Bioinformatics analysis was used to identify differentially expressed genes in septic mice heart and validate key genes and pathways. The correlation of protein–protein and protein–pathway was analyzed. Sequentially, the cecal ligament and puncture (CLP) was used to induce septic mice, followed by Serpine1 inhibitor treatment. Finally, the regulatory relationship of Yes-associated protein1 (YAP1), Serpine1, and caspase-3 was verified in LPS-exposed mouse cardiomyocytes.

**Results:**

Bioinformatic analysis found that Serpine1 expression is decreased in septic mice heart tissue and closely related to the HIPPO signaling pathway, while YAP1 is negatively correlated with apoptosis. *In vivo*, CLP induced a reduction of survival rate, cardiac dysfunction, and an increase in Serpine1 and Cleaved Caspase-3 expression, which could be reversed by a Serpine1 inhibitor. *In vitro*, LPS induced the mouse cardiomyocytes apoptosis, which could be reversed by Serpine1 inhibitor. Silencing YAP1 and Serpine1 reversed the LPS-induced increase in Serpine1 and Cleaved Caspase-3 expression, but silencing Serpine1 did not affect the LPS-induced YAP1 expression.

**Conclusion:**

Sepsis induced mouse cardiomyocytes apoptosis and cardiac dysfunction through activation of YAP1/Serpine1/caspase-3 pathway.

## 1 Introduction

Sepsis, a life-threatening medical condition, is characterized by a dysregulated immune response to infection or injury [1], which causes significant damage to various organ systems, including the cardiovascular system responsible for blood circulation [2]. Cardiac dysfunction is a common complication of sepsis, which is involved in cardiomyocyte apoptosis [3]. The mortality rate in patients with septic cardiac dysfunction (SCD) is as high as 50%. Therefore, it is of great significance to reveal the pathogenesis of SCD to provide intervention targets in clinical treatment.

By analyzing the GSE9667 dataset in the GEO database, we found that Serpine1 gene expression was significantly lower in the heart of cecal ligation and puncture (CLP) model mice than that of the sham model, and through the GO/Pathway analysis, we found that Serpin family E member 1 (Serpine1) was closely related to the Hippo pathway. Serpine1 is a member of the serine protease inhibitor superfamily, which could be secreted by cardiomyocytes [4] and is closely related to anti-apoptosis [5,6] and pro-apoptosis [7]. Serpine1 has been reported to regulate apoptosis signaling by the mediation of caspase-3 activation [8], which is an essential executor in apoptosis, and cleavage of caspase-3 has been regarded as a biomarker of cell apoptosis [9]. The cardiomyocyte apoptosis plays a pivotal role in promoting SCD [10]. However, whether Serpine1 is involved in cardiomyocyte apoptosis promoting SCD has not been reported.

The Hippo pathway was first identified in *Drosophila*, and it controls organ size by regulating cell proliferation and apoptosis [11]. Yes-associated protein1 (YAP1) is the main downstream effector of the Hippo pathway [12]. Silencing of YAP1 promotes the cell apoptosis [13]. Some evidence indicated that YAP1 with the ability to regulate the transcription of Serpine1 [14,15]. Therefore, we speculate that the activation of the YAP1/Serpine1/Caspase-3 signal pathway may contribute to cardiomyocyte apoptosis in promoting SCD.

In the present study, we explored the role of Serpine1 in the cardiac dysfunction of septic mice and LPS-induced apoptosis of mouse cardiomyocytes (HL-1) and the potential mechanism. Therefore, this study is helpful in showing that Serpine1 may serve as an intervention target for SCD in the future.

## 2 Materials and methods

### 2.1 RNA-seq of GSE9667 dataset from the GEO database

The raw data of GSE9667 dataset (sham group versus CLP with Min Dep group) were downloaded from the GEO database. Then, the data were aligned to the mouse genome (UCSC mm9) using TopHat v2.0.4 with default options and then assembled using Cufflinks v2.2.1. The differentially expressed genes were chosen based on fold change >2 and *p* < 0.05.

### 2.2 Gene ontology (GO) and pathway analysis

The top 21 differentiable expressed genes from the GSE9667 dataset were selected for functional enrichment analysis, which was performed based on GO resources and Kyoto Encyclopedia of Genes and Genomes resources. GO and pathway analysis were performed by using the Bioinformatics online tool (https://www.bioinformatics.com.cn). The significance cutoff for FDR was set at 0.05.

### 2.3 Experimental animals

The male, 8-week-old, C57BJ/6 mice weighing 25 ± 2 g (ENSIWEIER Biotechnology Co., Ltd, Chongqing, China) were chosen as experimental subjects. All the mice were kept in an SPF animal-keeping house and provided with enough sterile water and food. The animal experiment was approved by the Animal Ethics Committee of Chongqing University Central Hospital (Animal Experimental Ethical Inspection Form of Chongqing University Central Hospital No. 2300076).

### 2.4 CLP and sham operation

The mice who underwent CLP operation were anesthetized with 2% isoflurane before the procedure. Then, a surgical incision was made to expose the cecum, which was ligated approximately 1 cm from its end and punctured using a No. 22 needle. Sham-operated mice underwent the same operation as CLP mice but without ligation and puncture of the cecum.

### 2.5 Survival studies

Survival rates were analyzed using the GraphPad software. In brief, the mice were randomly assigned into the sham group, the CLP group, the CLP + 200 nM Diaplasinin (HY-122098, Med Chem Express, USA) group, and the CLP + 400 nM Diaplasinin group (20 samples/group). The mice in the sham group underwent a sham operation and the mice in the CLP group underwent a CLP operation. The mice in the CLP + 200 nM Diaplasinin group received intraperitoneal injections of 200 nM Diaplasinin per day. The mice in the CLP + 400 nM Diaplasinin group received intraperitoneal injections of 400 nM Diaplasinin per day. Survival curves were generated at 24-h intervals over a period of 7 consecutive days. Following the completion of this time frame, surviving mice were administered anesthesia using 2% isoflurane and subsequently euthanized via cervical dislocation. The collected data underwent analysis utilizing GraphPad software.

### 2.6 Echocardiography

The mice after CLP at day 1 were anesthetized with 2% isoflurane before ultrasonic echocardiography. The echocardiography (Philips TIS 0.8, Philips N.V.) and an RMV 707B transducer were utilized for this purpose. The diameter of the left ventricle during both diastole and systole was assessed from a short-axis perspective just below the mitral valve, toward the right side of the sternum. The values for left ventricular ejection fraction (LVEF) and fractional shortening (LVFS) were automatically calculated by echocardiography. After the echocardiography was done, the mice were killed via cervical dislocation immediately and the hearts were collected for subsequent immunohistochemistry (IHC) and Western blotting experiments.

### 2.7 IHC

The expression and distribution of Cleaved Caspase-3 in the heart tissues of mice were detected by IHC. In brief, the paraffin sections of the mouse heart were deparaffinized in xylene and in gradient alcohol solution (100% → 100% → 90% → 80% → 70%). The hydrogen oxide was used to block the endogenous peroxidase followed by sheep serum blocking and, then, incubated at 20°C for 2 h with polyclonal primary rabbit anti-Cleaved Caspase-3 antibody (Cat No. 25128-1-AP, dilution: 1:100, Proteintech Group, Wuhan, China). Then, sections were incubated with Biotin-conjugated affinipure Goat Anti-Rabbit IgG (dilution: 1:500, Cat No. SA00004-2, Proteintech Group) at 20°C for 30 min. Sequentially, the DAB substrate was used for staining. In the last step, sections were rinsed with tap water, stained with hematoxylin reagent, dehydrated with gradient alcohol solution (70% → 80% → 90% → 100% → 100%), cleared, and cover slipped.

### 2.8 Cell culture

The mouse atrial cardiomyocyte (HL-1 cell line) was purchased from Fan Tai Biotechnology Co., Ltd (Shanghai, China). The cells were cultured in HL-1 cell-specific medium (Cat No. CM-0605, Procell Co., Ltd, Wuhan, China) in a humidified incubator (5% CO_2_ at 37°C). The vitro experiments were performed until cells were cultured to achieve 70–80% confluence.

### 2.9 Measurement of cell viability (cell counting kit‐8 [CCK-8] assay)

The viability of HL-1 cells was measured by CCK-8 (Beyotime, Shanghai, China). In brief, HL-1 cells were seeded in 96-well microplate at a density of 5 × 10^3^ per well and then co-cultured with LPS (0, 0.1, 1.0, and 10.0 mg/l) for 24 h. Sequentially, CCK-8 reagent (10 µl) was added, followed by culturing at 37°C for 1 h. The optical density was detected at 450 nm in a micro-plate reader. The cell viability was calculated based on the optical density.

### 2.10 Transfection of siRNA-Serpine1, siRNA-YAP1, and siRNA-NC

The siRNA-Serpine1, siRNA-YAP1, and siRNA-NC were purchased from Shanghai Gene Chem Co., Ltd (Shanghai, China). The HL-1 cells were seeded in a six-well plate and, then, the transfection of siRNA-Serpine1 (50 nM), siRNA-YAP1 (50 nM), and siRNA-NC (50 nM) was performed by using the Lipofectamine™ 2000 regent (Thermo Fisher Scientific, USA). After 24 h, cells were exposed to 10.0 mg/l LPS. The expression of YAP1 protein in the cells was determined by Western blotting.

### 2.11 Flow cytometry analysis of cell apoptosis rate

HL-1 cells were seeded at 1 × 10^5^ cells per well into a six-well plate. When the cells were cultured to achieve 80% confluence, transfection with or without siRNA-Serpine1 for 24 h, then expose to 10.0 mg/l LPS for 24 h. The cells were washed with phosphate-buffered saline to remove ethylenediaminetetraacetic acid, re-suspended in a binding bluffer, and stained with Annexin V/PI (Beyotime Biotechnology, Shanghai, China) for 15 min in the dark. Fluorescence from 10,000 cells in the Annexin V–FITC and PI binding channels FL-1 (Annexin V-FITC) and FL-3 (PI) was quantified using FACScan and analyzed using Cellquest Pro. The apoptosis rate = percentage of early apoptotic cells + late apoptotic cells.

### 2.12 Western blotting

HL-1 cells were seeded into a six-well plate. When cells were cultured to achieve 80% confluence, transfection with or without siRNA-Serpine1 for 24 h, then expose to 10.0 mg/l LPS for 24 h. The protein was harvested from the mouse heart and HL-1 cells. 30 μg of protein product were electrophoresed in 10% sodium dodecyl sulfate polyacrylamide gel electropheresis gel, and then, the protein gel was transferred to a polyvinylidene fluoride (PVDF) membrane. Sequentially, the protein PVDF membrane was blocked with 5% no-fat milk for 1 h at room temperature, followed by incubating with primary antibodies against YAP1 (dilution: 1:1,000, Cat No. 13584-1-AP, Proteintech Group), Serpine1 (dilution: 1:1000, Cat No. 66261-1-Ig, Proteintech Group), Caspase-3 (dilution: 1:1,000, Cat No. 19677-1-AP, Proteintech Group), Cleaved Caspase-3 (dilution: 1:1,000, Cat No. 25128-1-AP, Proteintech Group), and GAPDH (dilution: 1:5,000, Cat No. 10494-1-AP, Proteintech Group) at 4°C overnight, and incubated with HRP anti-rabbit/mouse IgG antibody (1:5,000, ab288151/ab96879, Abcam) at room temperature for 2 h. In the last step, protein bands were visualized and analyzed with ChemiDoc™ XRS + with the Image Lab™ Software Gel Imaging System (Bio-Rad Laboratories).

### 2.13 Statistical analysis

**Figure 1:**
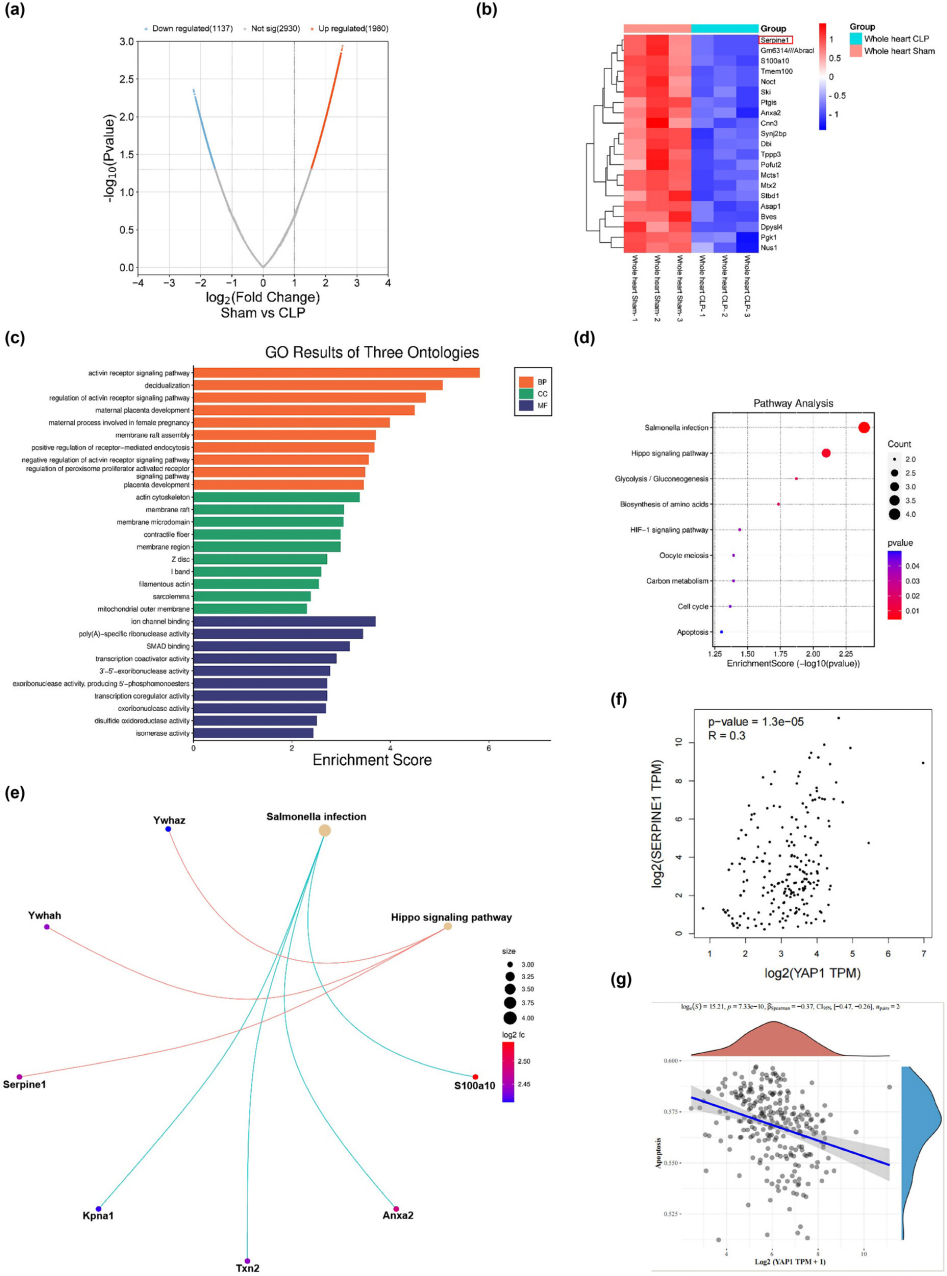
Bioinformatic analysis of Serpine1 in CLP mice heart and the correlation with pathway. (a) The volcano map of the GSE9667 dataset from the GEO database. (b) The heat map of top 21 differentiable expressed genes from the GSE9667 dataset. (c–e) The results of GO/pathway analysis of the top 21 differentiable expressed genes from the GSE9667 dataset. (f and g) Spearman correlation analysis was used to analyze the correlation between YAP1 and Serpine1 protein expression in the GEPIA database and the correlation between YAP1 gene and apoptosis in the TCGA database.


GraphPad Prism version 9.0 was used for all statistical analyses. The data were expressed as the mean ± standard deviation. Log-rank (Mantel–Cox) was performed to analyze the survival rate between groups. The unpaired *t*-test with two-tailed was performed for two groups comparison in Figure 1b–d. The comparison among groups was determined by the one-way analysis of variance followed by Dunnett’s multiple comparisons test. Statistical significance was indicated by *p*-value <0.05.

**Ethical approval:** The animal experiment was approved by the Animal Ethics Committee of Chongqing University Central Hospital, and performed in accordance with the Chongqing University Guidelines for Animal Care and Use (Animal Experimental Ethical Inspection Form of Chongqing University Central Hospital No. 2300076).

## 3 Results

### 3.1 The CLP decreased the expression of Serpine1, which is related to the Hippo signaling pathway and apoptosis in mice heart

The bioinformatics analysis was performed to identity the differentially expressed genes in septic mice heart from the GSE9667 dataset in the GEO database. The volcano map showed that there are 1,137 genes down-regulated and 1,980 genes up-regulated in mice heart of the sham group versus the CLP group (Figure 1a). The heat map showed that Serpine1 was significantly low expressed in the mice heart of the CLP group (Figure 1b). Sequentially, the GO/pathway analysis showed that Serpine1 is closely related to the HIPPO signaling pathway (Figure 1c–e). We used the GTEx sub-database to analyze the correlation between YAP1 and Serpine1 protein expression in the GEPIA database and found that YAP1 was positively correlated with Serpine1 protein expression (Figure 1f). At last, Spearman correlation analysis was used to analyze the correlation between the YAP1 gene and apoptosis in the TCGA database; we found that YAP1 was negatively correlated with apoptosis (Figure 1g).

### 3.2 CLP induces a decrease in survival rate, cardiac dysfunction, induction of Serpine1, and Cleaved Caspase-3 expression in mice

**Figure 2:**
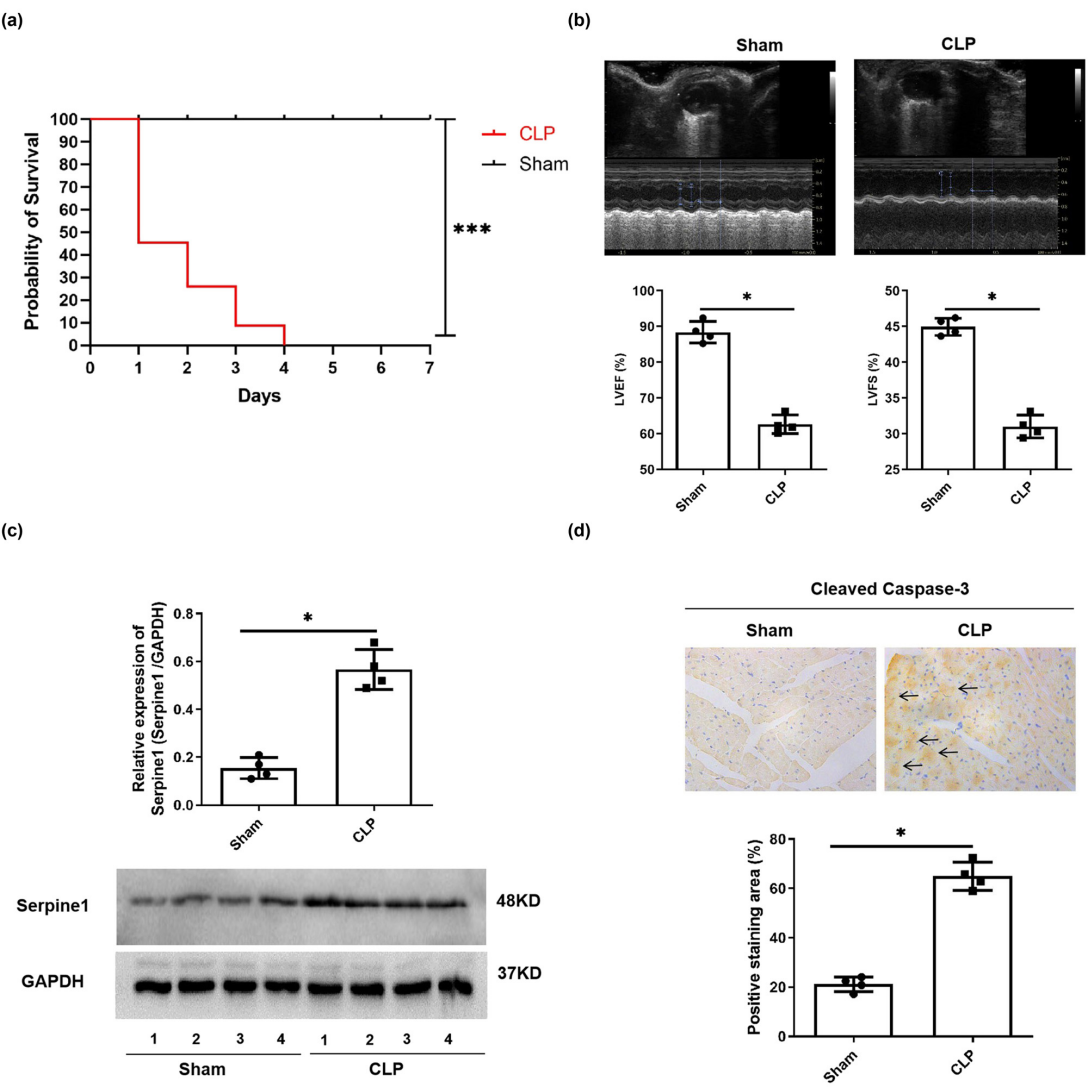
The effects of CLP on survival rate, cardiac function, and expression of Serpine1 and Cleaved Caspase-3 of mice. (a) The survival rates of mice in the sham and CLP groups were compared and analyzed. *N* = 20/group, ***p < 0.001. (b) The LVEF and LVFS of mice in the sham and CLP groups were compared and analyzed. *N* = 4/group, *p < 0.05. (c) The protein level of Serpine1 in the heart tissue of mice in the CLP and sham groups was detected by Western blot and analyzed. *N* = 4/group, *p < 0.05. (d) The distribution and expression of Cleaved Caspase-3 (as the arrows indicated; the brown signal is from the Cleaved Caspase-3 and the blue signal is from cell nuclei) in the heart tissue of mice in the CLP and sham groups were detected by IHC. *N* = 4/group, *p < 0.05.

The sepsis was induced by CLP, and the survival rates of mice in the sham group and the CLP group were compared. We observed that the survival rate of mice in the CLP group decreased from 100% to 45% on the first day, to 25% on the second day, to 5% on the third day, and to 0% on the fourth day, while the survival rate of mice in the sham group kept at 100% for 7 days. The difference in the survival rate of the CLP and sham groups was significant (Figure 2a, *p* < 0.001). The cardiac function of mice was determined, and the result showed that the LVEF and LVFS of the CLP group were significantly inhibited than that of the sham group (Figure 2b, *p* < 0.05). We also detected the expression of Serpine1 and Cleaved Caspase-3 in the heart tissue of mice in the CLP and sham groups. The result showed that CLP significantly increased the expression of Serpine1 (Figure 2c, *p* < 0.05) and the cleavage of caspase-3 (Figure 2d, *p* < 0.05).

### 3.3 Diaplasinin (Serpine1 inhibitor) reverses the decrease in survival rate, cardiac dysfunction, induction of Serpine1, and Cleavage of Caspase-3 induced by CLP

**Figure 3:**
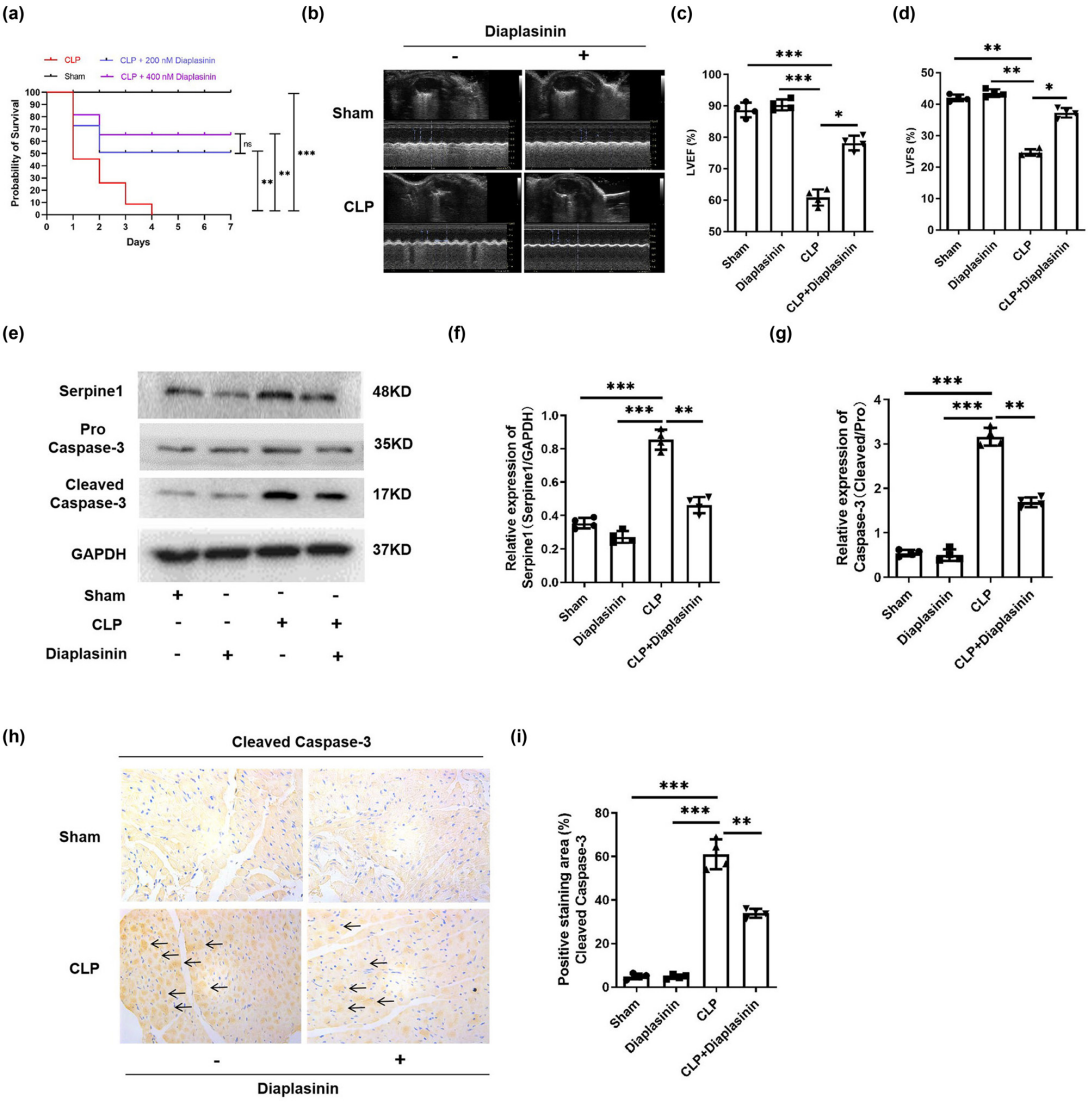
Inhibition of Serpine1 reverses the decrease of survival rate, cardiac dysfunction, induction of Serpine1, and cleavage of caspase-3 induced by CLP. (a) The survival rates of mice in the sham, CLP + 200 nM Diaplasinin, CLP + 400 nM Diaplasinin, and CLP groups were compared and analyzed. *N* = 20/group, **p < 0.01, ***p < 0.001. (b–d) The LVEF and LVFS of mice in the sham, Diaplasinin, CLP + Diaplasinin, and CLP groups were compared and analyzed. *N* = 4/group, *p < 0.05, **p < 0.01, ***p < 0.001. (e–g) The protein level of Serpine1 and Cleaved Caspase-3 in the heart tissue of mice in the sham, Diaplasinin, CLP + Diaplasinin, and CLP groups were detected by Western blot and analyzed. *N* = 4/group, **p < 0.01, ***p < 0.001. (h and i) The distribution and expression of Cleaved Caspase-3 (as the arrows indicated, the brown signal is from the Cleaved Caspase-3 and the blue signal is from cell nuclei) in the heart tissue of mice in the sham, Diaplasinin, CLP + Diaplasinin, and CLP groups were detected by IHC. *N* = 4/group, **p < 0.01, ***p < 0.001.

To explore the role of Serpine1 in CLP-induced sepsis, Diaplasinin (Serpine1 inhibitor) was intraperitoneally injected when CLP was performed on mice. We observed that 200 or 400 nM Diaplasinin could significantly alleviate CLP-induced decrease in survival rate (Figure 3a, *p* < 0.01); however, no significant difference in survival rate was observed between 200 and 400 nM Diaplasinin treatment group (Figure 3a, p *>* 0.05). Therefore, we chose 200 nM Diaplasinin for the follow-up experiment. The cardiac function of mice was determined, and the result showed that Diaplasinin significantly alleviated CLP-induced inhibition of LVEF and LVFS of mice (Figure 3b, *p* < 0.05). We also observed that Diaplasinin significantly reversed the increase of Serpine1 (Figure 3e and f, *p* < 0.01) and cleavage of caspase-3 (Figure 3e, g, h and i, *p* < 0.01) induced by CLP.

### 3.4 Silencing of Serpine1 alleviates the LPS-induced apoptosis of mouse cardiomyocyte

**Figure 4:**
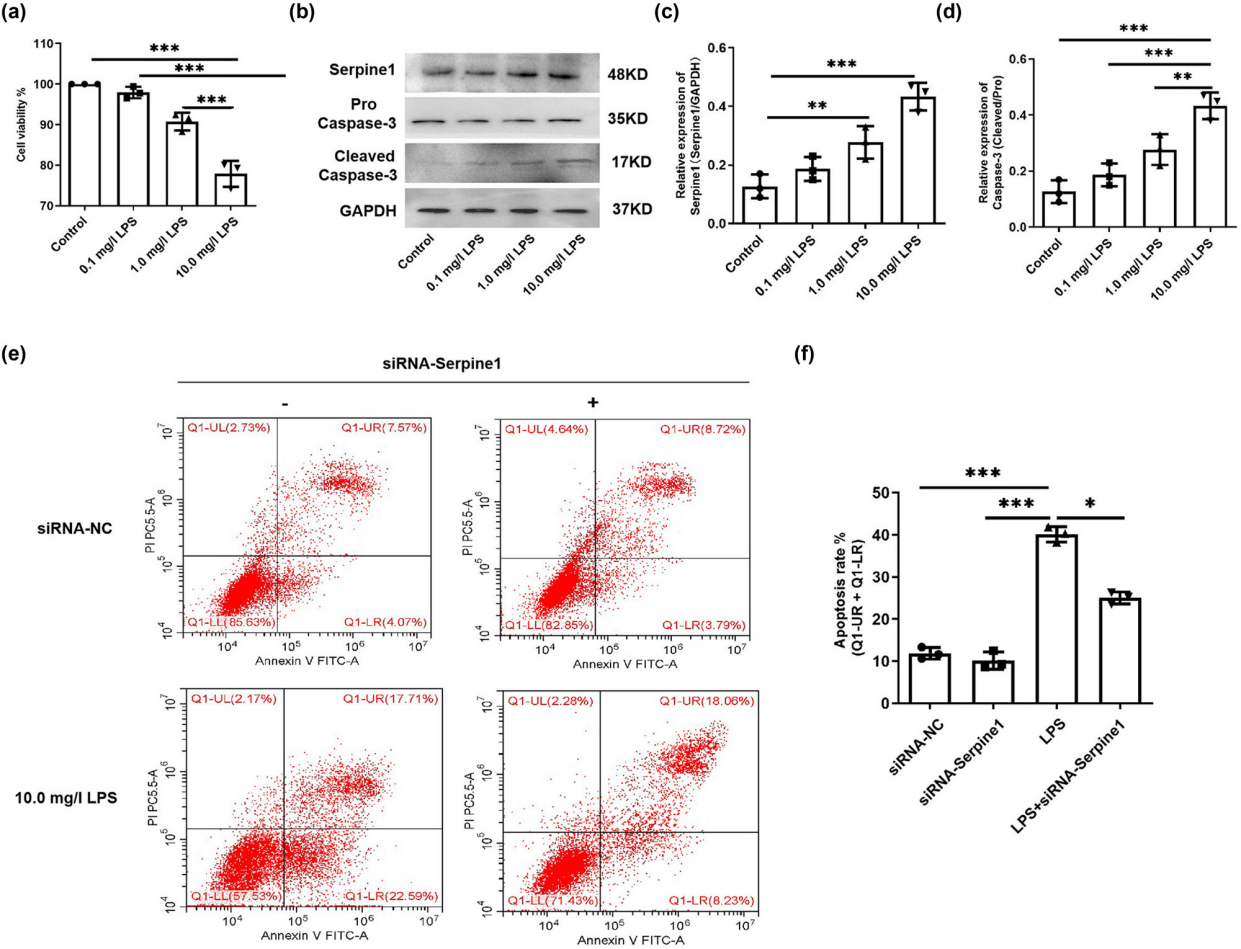
Silencing of Serpine1 alleviates the apoptosis of mouse cardiomyocytes induced by LPS. (a) The cell viability of HL-1 cells from the control (0), 0.1, 1.0, and 10.0 mg/l LPS treatment group was detected by CCK-8, *N* = 3, ***p < 0.001. (b–d) The protein level of Serpine1 and Cleaved Caspase-3 of HL-1 cells from the control (0), 0.1, 1.0, and 10.0 mg/l LPS treatment group was detected by Western blot and analyzed. *N* = 3, **p < 0.01, ***p < 0.001. (e and f) The apoptosis rate of HL-1 cells from the control, siRNA-Serpine1, LPS, and LPS + siRNA-Serpine1 group was detected by flow cytometry. *N* = 3, *p < 0.05, ***p < 0.001.

LPS was treated with mouse cardiomyocyte (HL-1 cell) to mimic the septic model *in vitro*. The result showed that LPS induced the inhibition of HL-1 cell viability in a concentration (0, 0.1, 1.0, 10.0 mg/l)-dependent manner (Figure 4a, all *p* < 0.01). Sequentially, we detected the expression of Serpine1 and Cleaved Caspase-3, and the result showed that LPS induced the inhibition of Serpine1 (Figure 4b and c, all *p* < 0.05) and cleavage of caspase-3 (Figure 4b and d, all *p* < 0.01) in a concentration (0, 0.1, 1.0, 10.0 mg/l)-dependent manner.

To explore the role of Serpine1 in LPS induced the decrease of HL-1 cell viability, the siRNA-Serpine1 was transferred into the HL-1 cells prior to LPS treatment, then, the apoptosis rate of HL-1 cells was detected by flow cytometry, and the result showed that silencing of Serpine1 could significantly reverse the 10.0 mg/l LPS-induced apoptosis of HL-1 cells (Figure 4e and f, *p* < 0.05).

### 3.5 LPS induces the cleavage of caspase-3 in promoting apoptosis through activation of YAP1/Serpine1 axis

**Figure 5:**
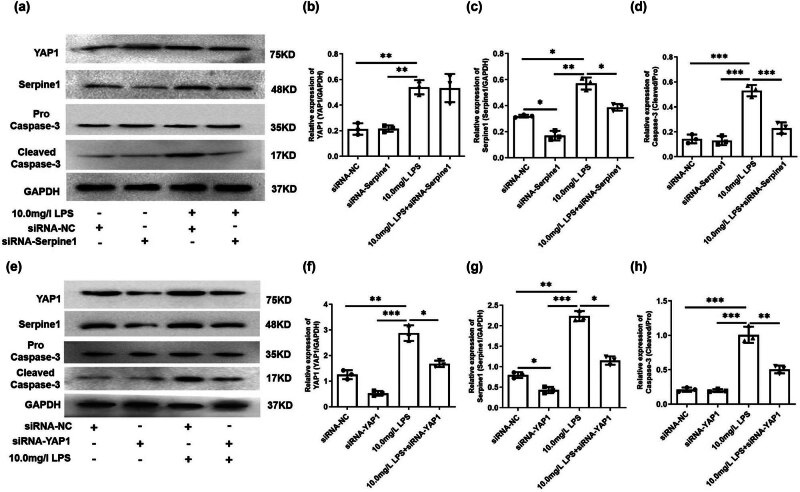
The YAP1/Serpine1 axis is involved in LPS induced the cleavage of caspase-3. (a–d) The protein level of YAP1, Serpine1, and Cleaved Caspase-3 of HL-1 cells from siRNA-NC, siRNA-Serpine1, LPS, and LPS + siRNA-Serpine1 group was detected by Western blot and analyzed. *N* = 3, *p < 0.05, **p < 0.01, ***p < 0.001. (e–h) The protein level of YAP1, Serpine1, and Cleaved Caspase-3 of HL-1 cells from the siRNA-NC, siRNA-YAP1, LPS, and LPS + siRNA-YAP1 groups was detected by Western blot and analyzed. *N* = 3, *p < 0.05, **p < 0.01, ***p < 0.001.

The YAP1 and Serpine1 were involved in the regulation of apoptosis; whether YAP1/Serpine1 plays a role in LPS-induced apoptosis of HL-1 cells needs to be further verified. The siRNA-Serpine1 was transferred into the HL-1 cells prior to the 10.0 mg/l LPS treatment, followed by expression of YAP1; Serpine1 and Cleaved Caspase-3 were detected. The result showed that LPS induced the increase of YAP1 (Figure 5a and b, *p* < 0.01) and Serpine1 expression (Figure 5a and c, *p* < 0.05) and cleavage of caspase-3 (Figure 5a and d, *p* < 0.001), and silencing of Serpine1 reversed the LPS induced increase of Serpine1 (Figure 5a and c, *p* < 0.05) and cleavage of caspase-3 (Figure 5a and d, *p* < 0.001). Moreover, we found that silencing of Serpine1 with no effect on LPS-induced YAP1 expression (Figure 5a and b, *p* > 0.05).

Next, we silenced YAP1 expression by transfection of siRNA-YAP1 into the HL-1 cells prior to the 10.0 mg/l LPS treatment; then, expression of YAP1, Serpine1, and Cleaved Caspase-3 were detected. The result showed that silencing of YAP1 (Figure 5e and f, *p* < 0.05) significantly reversed the LPS-induced increase in Serpine1 expression (Figure 5e and g, *p* < 0.05) and cleavage of caspase-3 (Figure 5e and h, *p* < 0.01).

## 4 Discussion

In this study, we found that CLP induced a reduction of survival rate, cardiac dysfunction, and an increase in Serpine1 expression and cleavage of caspase-3, which could be reversed by Serpine1 inhibitor treatment *in vivo*. *In vitro*, we demonstrated that LPS induced the cleavage of caspase-3 in promoting HL-1 cell apoptosis through activation of the YAP1/Serpine1 axis.

Myocardial injury is a significant outcome and a notable factor leading to mortality in instances of sepsis, an intense infection distinguished by a unique inflammatory reaction and posing a potential danger to the patient’s survival [16]. The main outcome of myocardial injury is cardiac dysfunction or failure, which is an important reason for the poor prognosis of sepsis [17]. Cardiac dysfunction is one of the major complications of sepsis, and SCD is considered a leading cause of death in sepsis [18]. In our investigation, we discovered that CLP markedly decreased the survival rate of mice and induced cardiac dysfunction (Figure 2a and b).

Abnormal apoptosis of cardiomyocytes is an integral and critical pathological basis underlying myocardial injury [19]. In the context of myocardial injury, abnormal apoptosis of cardiomyocytes can be triggered by a multitude of factors, such as ischemia [20], oxidative stress [21], and inflammation [22]. It has been reported that CLP or LPS-induced sepsis caused cardiomyocyte apoptosis [23,24]. CLP or LPS-induced sepsis resulted in acute cardiac injury through promoting apoptosis [25,26]. We also observed that CLP induced the cleavage of caspase-3 in the heart tissue of mice (Figure 2d) and LPS induced the increase of apoptosis (Figure 4f and g) and cleavage of caspase-3 (Figure 4b and d) in mouse cardiomyocyte.

Serpine1 also called plasminogen activator inhibitor-1 (PAI-1) plays an important role in the pathophysiology of sepsis [27]. Elevated Serpine1 level is related to a worse prognosis of sepsis [28]. Significant increases in Serpine1 expression were observed in mice or cells treated with either LPS [29] and CLP [30,31]. Chen et al. [32] found that Sepine1 was one of the drug target genes for Septic Cardiomyopathy based on bioinformatic analyses. Our bioinformatic study showed that the Sepine1 mRNA was down-regulated in the heart of CLP mice (Figure 1b); however, in our vivo and vitro study, we observed that CLP or LPS treatment induced the up-regulation of Serpine1 protein level (Figures 2c and 3b). The exact reason is unknown, maybe the differences in the copy of the disease model, RNA degradation, and errors by the experimental operator could be a response to the reason our experimental study is contrary to the data from the GSE9667 dataset. Many evidences show that PAI-1 is involved in the regulation of apoptosis [33]. It has been reported that genetic and pharmacological inhibition of Serpine1 led to apoptosis mainly mediated by caspase-9 activation in chronic myeloid leukemia CD34 cells [34]. However, silencing of Serpine1 could augment the apoptosis of gastric cancer cells [35]. Moreover, the inhibition of PAI-1 prevented the apoptotic and inflammatory actions in human umbilical vein endothelial cells [7]. PAI-1 deficient inhibited apoptosis, while restored PAI-1 levels, induced apoptosis in human pulmonary vascular smooth muscle cells [36]. Therefore, Serpine1 may play anti-apoptotic and pro-apoptotic roles in different cells. In our study, we found that silencing of Serpine1 alleviated the LPS-induced apoptosis and cleavage of caspase-3 *in vivo* (Figure 3i and j) and *in vitro* (Figures 4f, g and 5a, d).

The Hippo pathway plays a crucial role in the process of apoptosis. The transcription co-regulator YAP1 of the Hippo pathway can promote cell proliferation and inhibit the transcription of apoptosis-related genes [37]. Genetic inhibition of YAP1 decreases cell proliferation and increases apoptosis [13]. However, a previous study has reported that YAP1 is extensively activated and promotes tumor cell apoptosis [38]. Moreover, YAP protein was activated in HPMECs during LPS- and CHX-induced HPMEC apoptosis, while silencing of YAP protein attenuated the apoptosis induced by LPS plus CHX [39]. Therefore, YAP has a dual role of anti-apoptotic and pro-apoptotic effects. In our study, we found that LPS-induced YAP1 expression was elevated, while YAP1 knockdown partially reversed LPS-induced caspase-3 cleavage (Figure 5e), which demonstrated that YAP1 positively regulated LPS-induced apoptosis of mouse cardiomyocytes. Paradoxically, bioinformatic analysis found that YAP1 was negatively associated with apoptosis (Figure 1g), possibly because the TCGA database was based on data from cancer studies.

YAP and transcriptional co-activator with PDZ-binding motif (TAZ) are two homologous transcriptional coactivators, which have a very similar structure and sequence and can regulate cell proliferation, migration, differentiation, and apoptosis by interacting with the transcription factor, for example, transcriptional enhancer associate domain (TEAD) family members [40]. YAP/TAZ, the downstream effectors of the Hippo pathway, is involved in regulating cell apoptosis [41]. However, it has not reported the effect of YAP/TAZ on sepsis-induced apoptosis of cardiomyocytes. Therefore, further research should investigate whether the functions of YAP/TAZ are the same or different in sepsis or complement or overlap in some way in the future.

YAP regulates PAI-1 expression and secretion, and the knockdown of YAP leads to reduced PAI-1 transcript abundance [15]. The elevated concentration of PAI-1 in the blood could indicate YAP activation [42]. Silencing of YAP or transcriptional co-activator with PDZ-binding motif significantly impaired TGFβ-mediated Serpine1 expression [14]. In our study, the bioinformatic analysis found that YAP1 expression was positively correlated with Serpine1 protein expression (Figure 1f). *In vitro* study, we verified that Serpine1 expression was positively mediated by YAP1 in LPS-exposed mouse cardiomyocytes (Figure 5).

## 5 Conclusions

In summary, we demonstrated that Serpine1 is responsive to sepsis-induced cardiomyocyte apoptosis and cardiac dysfunction in mice, and the activation of the YAP1/Serpine1/Caspase-3 signaling pathway is involved, which is expected to provide a potential target for clinical intervention of cardiac dysfunction induced by sepsis.

## 6 Limitations of the study

In this study, we discovered that the outcomes of the bioinformatics analysis were contrary to the Serpine1 experiment, and we were incapable of determining the genuine cause of this. We analyzed that there might be a discrepant correlation between RNA expression and protein translation in sepsis. Additionally, the variance in the experimental duration after CLP (24 vs 48 h) could also result in disparities in Serpine1 expression. We intend to further explore whether YAP1/Serpine1 expression is time dependent in future experiments.
